# Identification of Di/Tripeptide(s) With Osteoblasts Proliferation Stimulation Abilities of Yak Bone Collagen by *in silico* Screening and Molecular Docking

**DOI:** 10.3389/fnut.2022.874259

**Published:** 2022-05-30

**Authors:** Yongkai Chen, Yujie Guo, Yusi Liu, Chunhui Zhang, Feng Huang, Lingyun Chen

**Affiliations:** ^1^Institute of Food Science and Technology, Chinese Academy of Agricultural Sciences, Beijing, China; ^2^Department of Agricultural, Food and Nutritional Science, University of Alberta, Edmonton, AB, Canada; ^3^Wageningen Food and Biobased Research, Wageningen University and Research, Wageningen, Netherlands

**Keywords:** yak bone collagen, di/tripeptides, osteoblasts proliferation, *in silico* screening, molecular docking

## Abstract

Endothelial protein C receptor (EPCR), cannabinoid receptor 2 (CBR2), and estrogen receptor α (ERα) play vital roles in osteoblasts proliferation. Also, collagen peptides have osteoblasts proliferation stimulation abilities, and di/tri-peptides could be absorbed by the intestine more easily. This study obtained three di/tripeptides with potential osteoblasts proliferation stimulation abilities of yak bone collagen, namely, MGF, CF, and MF, by *in silico* screening. Results suggested that these three peptides exhibited good absorption, distribution, metabolism, excretion, and toxicity (ADMET) properties. They also had strong affinities with EPCR, CBR2, and ERα, and the total -CDOCKER energy (-CE) values were 150.9469, 113.1835, and 115.3714 kcal/mol, respectively. However, further Cell Counting Kit-8 (CCK-8) assays indicated that only MGF could significantly (*P* < 0.05) stimulate osteoblasts proliferation at 0.3 mg/ml. At the same time, the proliferating index (PI) of the osteoblasts treated with MGF increased significantly (*P* < 0.05), and the alkaline phosphatase (ALP) activity decreased highly significantly (*P* < 0.01). In summary, MGF exhibited the potential to be an effective treatment for osteoporosis.

## Introduction

Osteoporosis is a systemic skeletal disease characterized by low bone mass and destruction of bone microstructure, which will result in a high risk of bone fragility and susceptibility to fracture (1, 2). About 590 million people over the age of 60 suffer from osteoporosis worldwide (3). The situation may worsen with the increasing aging of the world population, resulting in an enormous economic and social burden (4). Previous studies have demonstrated that it is an effective method for improving osteoporosis with bone collagen peptides (5–7).

Researchers have confirmed that osteoblasts proliferation could increase the number of osteoblasts and then contribute to the improvement of osteoporosis (8). Recent reports have shown that peptides derived from food could stimulate osteoblasts proliferation *via* epidermal growth factor receptor (EGFR) (9, 10). Ye et al. (11) reported that yak bone collagen peptides (YBCPs) could promote osteoblasts proliferation by inducing EGFR dimerization. Except for EGFR, receptors such as EPCR, CBR2, and ERα can also influence osteoblasts proliferation. For instance, activated protein C (APC) can stimulate osteoblasts proliferation through binding to EPCR (12); miR-187-3p can promote osteoblastic precursor cells proliferation by regulating CBR2 expression (13); also, estradiol can promote osteoblasts proliferation by ERα-mediated Wnt/β-catenin signal pathways (14). However, their potential to be the receptors of peptides with osteoblasts proliferation stimulation abilities has been seriously ignored.

Compared with other oligopeptides (<hexapeptides), di/tripeptides usually have a higher absorption level (15). Matsui (15) reported that di/tripeptides could be transported through H^+^-coupled peptide transporter 1 (PepT1), and their transportability may be 1,000-fold higher than pentapeptides. Besides, di/tripeptides are more stable against digestive enzymes’ degradation because fewer peptide bonds make them hard to be recognized by the gastrointestinal protease (16). There is more research showing that di/tripeptides have broad physiological activities. For example, dipeptide VY can significantly inhibit the activity of the angiotensin-converting enzyme (ACE) (17); tripeptide WIR has a potential anti-Alzheimer’s disease effect (18); and dipeptide YL exhibits antidepressant-like activities in mice (19). However, surprisingly less research was on di/tripeptides with osteoblasts proliferation stimulation abilities. The possible reason was that most studies on anti-osteoporosis peptides still followed the traditional methods (a process involving preparation, isolation, and characterization), requiring much labor and costs (20–22). *In silico* methods may increase the screening efficiency and avoid the weakness of traditional approaches to some extent (23).

Yak is a unique livestock animal on the Qinghai-Tibetan Plateau (24). Yak bones, rich in collagen and minerals, have been used to strengthen bones in Tibetan medicine from ancient times (25). Our previous studies have demonstrated that polypeptides from yak bone collagen could promote osteoblasts proliferation (11). To identify di/tripeptides with osteoblasts proliferation stimulation abilities of yak bone collagen, *in silico* screening and molecular docking were performed. First, the sequence of the α_1_ and α_2_ chains of yak collagen-I was downloaded and digested virtually. Subsequently, di/tripeptides with good ADMET properties and potential proliferation stimulation abilities were obtained through bioactivity prediction, ADMET prediction, and molecular docking targeted on EPCR, CBR2, and ERα. Then, the proliferation stimulation abilities of these peptides were verified by CCK-8, cell cycle, and ALP assays. Moreover, the interaction mechanisms between di/tripeptides and targets were analyzed *via* molecular docking results.

## Materials and Methods

### Materials and Reagents

MC3T3-E1 cell lines and differentiation-induced medium were provided by Procell Life Science and Technology Co., Ltd. (Wuhan, China). Fetal bovine serum (FBS) was purchased from Zhejiang Tianhang Biotechnology Co., Ltd. (Beijing, China). Penicillin-streptomycin (P/S) was obtained from Yuan Ye Biotechnology Co., Ltd. (Beijing, China); 0.25% trypsin/EDTA and α-Minimum Essential (α-MEM) were purchased from Epsilon Technology Co., Ltd. (Shanghai, China). ALP assay kit was provided by Beyotime Biotechnology Co., Ltd. (Shanghai, China). CCK-8 reagent was purchased from Beijing Solarbio Biotechnology Co., Ltd. (Beijing, China). Propidium iodide was obtained from Biotopped Biotechnology Co., Ltd. (Beijing, China).

### Methods

#### Enzymolysis Simulation of Yak Bone Collagen

Collagen-I is the primary type of animal bone collagen with a triple-helical structure consisting of two α_1_ chains and one α_2_ chain (26). The sequence of the α_1_ chain (NCBI accession number: ELR60286) and α_2_ chain (NCBI accession number: ELR46121) (27) of yak collagen-I was downloaded from the NCBI database.^1^ Three common proteases in food laboratories, namely, proteinase K, pepsin, and trypsin, were used for enzymolysis simulation by the program ExPASy PeptideCutter.^2^ The protease with most cutting sites will be used to obtain di/tripeptides.

#### Bioactivities Prediction

The bioactivities of di/tripeptides released from yak collagen-I were predicted by the online tool PeptideRanker^3^ (28). The peptides with prediction scores ≥ 0.5 were used for further study.

#### Absorption, Distribution, Metabolism, Excretion, and Toxicity Prediction

According to the instruction, the ADMET properties of these peptides were predicted by Discovery Studio 2019 (BIOVIA). In detail, bad valencies were fixed after sketching the primary structures of peptides. Then, the “aqueous solubility,” “cytochrome P4502D6 inhibition,” “hepatotoxicity,” and “human intestinal absorption (HIA)” of peptides with prediction scores ≥ 0.5 were predicted *via* ADMET predictors.

#### Molecular Docking

The procedure of molecular docking was based on the method adopted by Vidal-Limon et al. (29), with a slight modification. The crystal structures of EPCR (PDB ID: 1LQV) (30), CBR2 (PDB ID: 6PT0) (31), and ERα (PDB ID: 1X7R) (32) were obtained from the Protein Data Bank (PDB).^4^ Their structures were constructed with protein cleaning and preparation. The docking sites were set at the binding position of the original ligands. For peptides, CHARMm forcefield was imputed and then minimized by the Smart Minimizer algorithm. The max steps were set as 2,000, and the RMS gradient was 0.01. After these pretreatments, the CDOCKER protocol of Discovery Studio 2019 (BIOVIA) was used for molecular docking.

#### Synthesis of Di/Tripeptides

Peptides MGF, CF, and MF were synthesized chemically by Beijing Protein Innovation Co., Ltd. (Beijing, China) and were characterized by the liquid chromatography-mass spectrometry (HPLC-MS) method. The HPLC conditions were as follows: The inject volume was 10 μl, and the detection wavelength was 220 nm. A flow rate of 1.0 ml/min was utilized on a Thermo Fisher HPLC equipped with a Kromasil 100-5C_18_ (4.6 mm, 250 mm, 5 μm). Buffer A was 0.1% trifluoroacetic acid (TFA) in acetonitrile, and buffer B was 0.1% TFA in water. Elution started with 20% buffer A followed by a gradient to 45% buffer A for 20 min and then a gradient to 80% buffer A for 0.1 min. The purity of all synthesized peptides was more than 98%.

#### Cell Counting Kit-88 Assay

MC3T3-E1 cells were cultured in α-MEM medium containing 1% P/S and 10% FBS and incubated at 37^°^C in a humidified atmosphere with 5% CO_2_. The procedure of cell proliferation assays was based on the method adopted by Che et al. (33), with a slight modification. MGF, CF, and MF were dissolved in the α-MEM medium containing 1% FBS and 1% P/S. Then, MC3T3-E1 cells were seeded in a 96-well plate at a density of 2 × 10^3^/well. After 24 h of adherence and 24 h of starvation, the cells were treated with peptides MGF, CF, and MF at concentrations ranging from 0 to 1.0 mg/ml for 72 h, respectively. The relative proliferative rate was measured according to the instructions provided by the CCK-8 reagents supplier (Solarbio, Beijing, China).

#### Cell Cycle Assay

The procedure of cell cycle assays was based on the method adopted by Liu et al. (5), with a slight modification. MC3T3-E1 cells were seeded in a 6-well plate and treated with peptide MGF (0.30 mg/ml) and CF (1.00 mg/ml) for 72 h, respectively. Then, the cells were collected and washed twice with precooling PBS. Afterward, the cells were fixed with 75% ethanol for 12 h and washed twice with precooling PBS. Their DNA contents were stained with propidium iodide and analyzed by the flow cytometer (CytoFLEX, Beckman, United States). The proliferation index was calculated based on the following equation (34):


(1) $$\mathrm{Proliferation}\mathrm{index}\left(\text{PI}\right)\left(\%\right)=\frac{\text{S}+\mathrm{G2}/M}{\mathrm{G0}/\mathrm{G1}+\text{S}+\mathrm{G2}/M}\times 100$$

#### Alkaline Phosphatase Activity Assay

The procedure of ALP activity assays was based on the method adopted by Zhu et al. (7), with a slight modification. Peptides MGF and CF were dissolved in the differentiation-induced medium containing 1% FBS and 1% P/S. MC3T3-E1 cells were treated with peptide MGF (0.30 mg/ml) and CF (1.00 mg/ml) for 120 h, respectively. Their ALP activities were measured according to the instruction of the ALP assay kit supplier (Beyotime, Shanghai, China). The absorbance was detected at 405 nm using a microplate reader (Eon, BioTek, United States).

#### Statistical Analysis

All experiments were repeated at least three times. The data were presented as mean value ± standard deviation and analyzed by one-way ANOVA of SPSS 22.0 software (SPSS Inc.). Significant level was set at *P* < 0.05 and *P* < 0.01.

## Results and Discussion

### Sequence Analysis of Yak Bone Collagen and Enzymolysis Simulation

**FIGURE 1:**
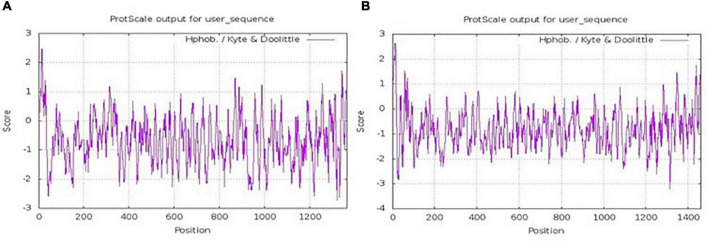
Hydrophobicity distribution of the amino acids of α_1_ chain **(A)** and α_2_ chain **(B)** of yak collagen-I.

Based on the sequence information of yak collagen-I provided by the NCBI database^5^ (Supplementary Figure 1), the α_1_ chain was found to consist of 1,459 amino acids, and the wealthiest amino acids were glycine (26.7%), proline (19.1%), and alanine (9.8%). Similarly, the α_2_ chain consisted of 1,366 amino acids, and the wealthiest amino acids were also glycine (27.9%), proline (17.2%), and alanine (9.0%). The sequence of both α_1_ chain and α_2_ chain was consistent with the typical collagen sequence (Glycine -X-Y)*_*n*_*, where X was usually a proline (35). The hydrophilicity and hydrophobicity of the α_1_ chain and α_2_ chain were analyzed by ProtScale^6^ (36). As shown in Figure 1A, the highest and lowest amino acid scale values of the α_1_ chain were 2.633 and -3.200, respectively. Also, the values of the α_2_ chain were 2.467 and -2.733, respectively (Figure 1B). The larger positive value indicated the greater hydrophobicity, while the smaller negative value indicated the greater hydrophilicity (37). This result suggested that unwinding yak bone collagen-I could be soluble in water and hydrolyzed by proteases, which was consistent with previous studies (21, 38, 39).

Many proteases have been used to prepare bone collagen peptides, such as papain, trypsin, and pepsin. It was a prerequisite to choose a protease with most cutting sites because more di/tripeptides could be obtained. Three common proteases in food laboratories, namely, trypsin, proteinase K, and pepsin, were selected to predict their yak bone collagen-I hydrolysis capacities. Pepsin, a kind of aspartic protease rich in polar and aspartic acid residues (40), has different enzyme cutting sites at different pH values. Therefore, the hydrolysis capacity of pepsin was considered, respectively, at pH > 2 and pH 1.3. According to the data given in Table 1, both α_1_ chain and α_2_ chain of yak collagen-I could be hydrolyzed by these three proteases. For the α_1_ chain, cleavage sites ranged from 87 to 425. Pepsin (pH 1.3) had the lowest hydrolysis ability, while proteinase K had the highest hydrolysis ability. Similarly, the α_2_ chain could also be hydrolyzed more completely (with 417 cleavage sites) by proteinase K than the other two proteases. Hence, proteinase K was used to obtain di/tripeptides.

**TABLE 1:** The cutting sites number of proteases.

	Number		
Protease	α_1_ chain	α_2_ chain	Total
Proteinase K	425	417	842
Trypsin	119	116	235
Pepsin (pH > 2)	115	127	242
Pepsin (pH 1.3)	87	104	191

### Bioactivity Prediction for Di/Tripeptides

As indicated in Supplementary Table 1, after the enzymolysis simulation by proteinase K, 83 di/tripeptides were derived from the α_1_ chain, and 79 di/tripeptides were derived from the α_2_ chain of yak bone collagen-I. However, 48 peptides were repeated. So, the total number of di/tripeptides was 114. PeptideRanker, an online prediction tool, was used to predict the potential bioactivity of peptides. The prediction scores ranged from 0.0 to 1.0, where “0.0” indicates unlikely, and “1.0” indicates highly likely (41). Using PeptideRanker, Ding et al. (42) identified antioxidant peptides YSSPIHIW (0.74), ADLYNPR (0.65), and HYDSEAILF (0.53) from pea protein. Liu et al. (43) identified α-glucosidase inhibitory peptides KVIISAPSKDAPMF (0.50), SQHISTAGMEASGTSN MKF (0.51), and STFQQMW (0.77) from Changii Radix. Yu et al. (44) identified peptides ADM (0.52) and ADW (0.82) from Oncorhynchus mykiss nebulin as bitter taste receptor blockers. We believed that peptides with prediction scores ≥ 0.5 were worth studying in this work. The molecular weight and bioactivity prediction score of these 114 peptides were included in Supplementary Table 1. There were 41 peptides with bioactivity prediction scores ≥ 0.5, and they would be used for ADMET predictions and molecular docking.

### Absorption, Distribution, Metabolism, Excretion, and Toxicity Prediction and Molecular Docking

It is crucial to predict the ADMET properties in the search for lead compounds since some compounds may have poor aqueous solubility, and others may be toxic or have poor absorbency. These unexpected properties can lower research assurance and increase the overall program cycle and costs. Therefore, four ADMET properties of peptides, namely, aqueous solubility, cytochrome P4502D6 inhibition, hepatotoxicity, and HIA, were predicted by ADMET predictors of Discovery Studio 2019 (BIOVIA). Aqueous solubility is an important index that affects small peptides’ absorbability. Small peptides with good aqueous solubilities tend to have high biological availability (45). As shown in Supplementary Table 2, there were 16 dipeptides and 25 tripeptides for ADMET predictions in total. They had a similar or better aqueous solubility prediction compared with some reported bioactive di/tripeptides, such as ACE and DPP-IV inhibitory peptides ADF, MIR, and FGR from egg proteins (46). This endowed them with good drug-like properties. As an essential part of Phase-I metabolism, cytochrome P4502D6 could oxidize xenobiotics to increase their excretion from the body (47). The results suggested that all peptides could assimilate in Phase-I metabolism and have no drug-drug interactions since they did not exhibit cytochrome P4502D6 inhibition effects. This property was consistent with DMG, an ACE inhibiting tripeptide from soy proteins reported by Zhao et al. (48). However, 32 peptides showed hepatotoxicity and poor HIA (HIA > 1). In this case, the remaining 7 dipeptides (MF, CF, GF, ML, SF, GY, MA) and 2 tripeptides (MGF and PGF) with good ADMET properties were used for CDOCKER docking. -CE value, having a positive relationship with the stability of ligand-receptor complexes (14), was used to evaluate the affinity between peptides and receptors (EPCR, CBR2, and ERα). According to data given in Supplementary Table 2, the total -CE value ranged from 97.561 to 150.9469 kcal/mol. Tripeptide MGF had the biggest total -CE value with 150.9469 kcal/mol. There was little difference in the total -CE of dipeptide MF (113.1835 kcal/mol), CF (115.3714 kcal/mol), and GY (113.6556 kcal/mol). GY (HIA = 1), however, had a poorer HIA prediction compared with CF (HIA = 0) and MF (HIA = 0). Therefore, we speculated that MGF, CF, and MF had good ADMET properties and potential osteoblasts proliferation stimulation abilities.

### Effect of Peptides on MC3T3-E1 Cells Proliferation

**FIGURE 2:**
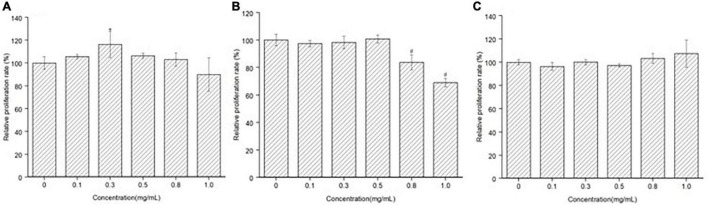
Effect of peptide MGF, CF, and MF on MC3T3-E1 cells’ proliferation. **(A)** Group treated with MGF for 72 h. **(B)** Group treated with CF for 72 h. **(C)** Group treated with MF for 72 h. *P < 0.05, ^#^*P* < 0.01.

The effect of MGF, CF, and MF on MC3T3-E1 cells proliferation was determined by the CCK-8 method. MGF had a significant (*P* < 0.05) proliferation stimulation ability at 0.30 mg/ml and exhibited good concentration-dependent effects (Figure 2A). CF, however, had a poor proliferation stimulation ability, and it could highly significantly (*P* < 0.01) suppress MC3T3-E1 cells proliferation at 0.80 and 1.00 mg/ml (Figure 2B). In the meantime, MF had no stimulation or suppression activity at concentrations ranging from 0 to 1.00 mg/ml, indicating that it may be a false-positive result (Figure 2C). Therefore, MGF and CF were used for further studies.

### Effect of Peptides on MC3T3-E1 Cells’ Proliferating Index and Alkaline Phosphatase Activity

**FIGURE 3:**
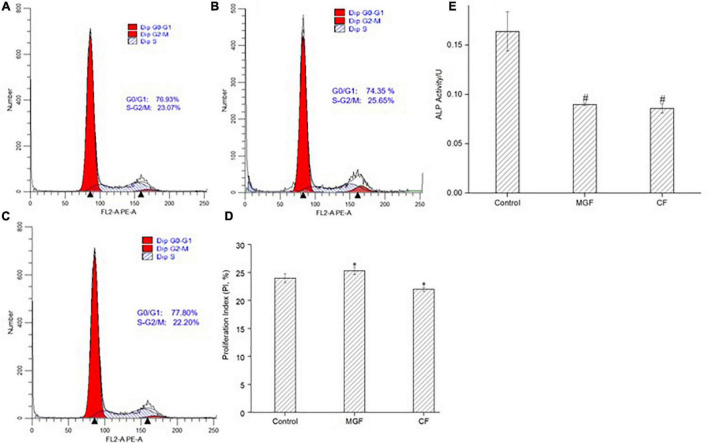
Effect of peptide MGF and CF on MC3T3-E1 cells’ PI and ALP activity. **(A)** Control group. **(B)** Group treated with MGF for 72 h. **(C)** Group treated with CF for 72 h. **(D)** PI of MC3T3-E1 cells treated with MGF and CF. **(E)** ALP activity treated with MGF and CF for 120 h. *P < 0.05, ^#^*P* < 0.01.

The effect of MGF and CF on MC3T3-E1 cells’ PI was further investigated by the flow cytometer. As indicated in Figures 3A,B,D, the PI of MC3TE-E1 cells significantly increased (*P* < 0.05) after treatment with MGF for 72 h, suggesting that the proportion of cells in the division stage increased. So, the osteoblasts proliferation stimulation effects of yak bone collagen peptides may attribute to the transformation of the cell cycle. This conclusion was consistent with the previous studies conducted by Liu et al. (5) since their studies also showed that the G1 phase of osteoblasts treated with bovine bone collagen peptides significantly decreased. Similarly, CF exhibited an opposite effect on the PI of osteoblasts (Figures 3A,C,D). ALP activity is the marker of early osteogenic differentiation (49). As shown in Figure 3E, the ALP activities of MC3T3-E1 cells treated with MGF and CF for 120 h both highly significantly (*P* < 0.01) decreased, which was different from other studies (7, 10). This result indicated that MGF might have a long-term proliferation-promoting effect since cells in the division stage have a poor differentiation ability. Adenosine 30,50-cyclic monophosphate (cAMP) and guanosine 30,50-cyclic monophosphate (cGMP) signaling pathways have an opposite effect on cell proliferation and differentiation. Zhang et al. (50) found that the elevation of intracellular cAMP could enhance bone morphogenetic protein (BMP) action and increase the ALP activity of osteoblastic cells in experimental animals. Therefore, the long-term proliferation-promoting effect of MGF may attribute to the concentration of intracellular cAMP being lower than cGMP. CF, however, could also suppress the ALP activities of osteoblasts, indicating that it might be the antagonist of these receptors, which was consistent with the CCK-8 and cell cycle assays.

### Molecular Interaction Mechanism of MGF, CF, and Receptors

The above results show that MGF and CF had an opposite effect on osteoblasts proliferation. To elucidate the reasons for this difference, molecular docking was performed to investigate the binding mode of MGF, CF, and receptors. Their molecular interaction mechanism was displayed, respectively, in the form of 2D images.

**FIGURE 4:**
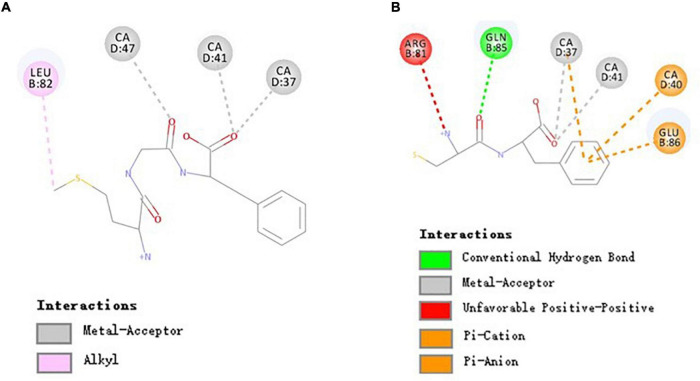
Molecular interactions of MGF, CF, and EPCR. **(A)** Molecular interactions of MGF and EPCR. **(B)** Molecular interactions of CF and EPCR.

The binding of peptides and EPCR could be Ca^2+^ dependent since Ca^2+^ may help position peptides to facilitate non-bond interactions with EPCR. As shown in Figure 4A, there were 3 common non-bond interactions of peptides and EPCR, namely, hydrogen bonds, electrostatic interactions, and hydrophobic interactions. The non-bond interaction between MGF and residue Leu82 of EPCR was alkyl interaction. At the same time, CA37, CA41, and CA47 formed metal-acceptors with MGF. CF was bound to EPCR by hydrogen bonds, pi-anion, and pi-cation interactions. Gln85 formed a hydrogen bond with CF, and Glu86 formed a pi-anion interaction. CA37 and CA40 made contact with CF by pi-cation interactions. The metal-acceptor interactions between CA37, CA41, and CF were also important. Unfavorable positive-positive interaction, however, was formed between Arg81 and CF (Figure 4B).

The EPCR is a multifunctional and multiligand receptor expressed highly in the endothelium lining blood vessels (51, 52). It can activate protein C (PC) to form activated protein C (APC, a signaling molecule downregulating thrombin generation) in a Ca^2+^-dependent manner (30, 53). However, an essential function of EPCR has been largely ignored in recent years. Studies conducted by Kurata et al. (12) showed that APC could promote osteoblasts proliferation through activating p44/42 MAP kinase by binding to EPCR. We found that some di/tripeptides, such as MGF, could have a similar effect with APC in this study. Moreover, the activity differences between MGF and CF may attribute to the different binding residues.

### Molecular Interaction Mechanism of MGF, CF, and Cannabinoid Receptor 2

**FIGURE 5:**
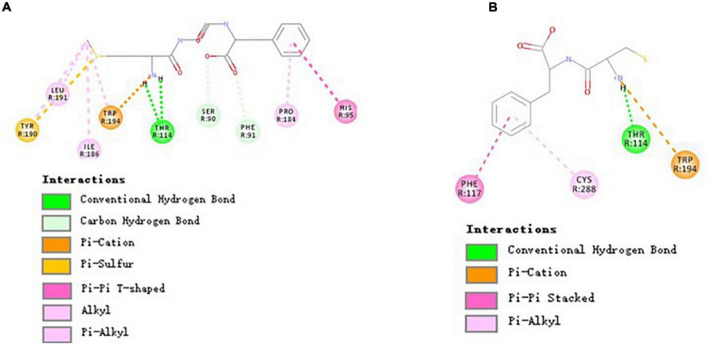
Molecular interactions of MGF, CF, and CBR2. **(A)** Molecular interactions of MGF and CBR2. **(B)** Molecular interactions of CF and CBR2.

As shown in Figure 5A, three residues (i.e., Ser90, Phe91, and Thr114) of CBR2 formed 4 hydrogen bonds with MGF (Thr114 formed 2 hydrogen bonds). In addition, the interactions of Tyr190, Tyr194, and MGF were pi-sulfur and pi-cation interactions, respectively. His95, Pro184, Ile186, Tyr190, Leu191, and Trp194 were bound with MGF by hydrophobic interactions. CF was bound to CBR2 by hydrogen bonds (Thr114), pi-cation interactions (Trp194), pi-pi stacked interactions (Phe117), and pi-alkyl interactions (Cys288) (Figure 5B).

Cannabinoid receptors are essential parts of the endocannabinoid system. There are two types of cannabinoid receptors, namely, cannabinoid receptor 1 (CBR1) and CBR2, in the human body (54). Drugs targeted in CBR2 may treat many disorders and avoid the psychiatric side effects of CBR1 (55). HU-308, a CBR2-specific agonist, can alleviate the Ti-induced decrease in osteoblast survival, mineralization capability, ALP, and osteocalcin activity (56). In this study, we found that both MGF and WIN 55,212-2 (a CBR2-specific agonist) could bind with residues Phe91, His95, Pro184, and Ile186 of CBR2 (31). Also, there were two same interaction sites (i.e., Thr114 and Trp194) of MGF and CF. Xing’s research (31) showed that Trp194, Phe117, and Trp258 of CBR2 played essential roles in distinguishing agonist (WIN 55,212-2) from the antagonist (AM10257), indicating that the peptides may have a different activation mechanism of CBR2. The result suggested that Thr114 and Trp194 played an essential role in the peptide-binding process, while other binding sites may determine the activity difference.

### Molecular Interaction Mechanism of MGF, CF, and Estrogen Receptor α

**FIGURE 6:**
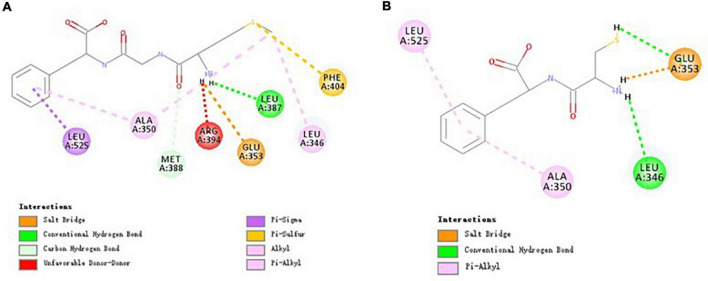
Molecular interactions of MGF, CF, and ERα. **(A)** Molecular interactions of MGF and ERα. **(B)** Molecular interactions of CF and ERα.

The results of Figure 6A suggested that MGF formed hydrogen bonds with Glu353 (also attractive charge interaction), Leu387, and Met388. The interactions of Leu346, Ala350, Phe404, Leu525, and MGF were maintained by hydrophobic interactions and pi-sulfur interaction. CF formed 3 hydrogen bonds with Glu353 (also attractive charge interaction) and Leu346. It also formed 2 pi-alkyl interactions with Ala350 and Leu525. All these residues were included by the MGF binding (Figure 6B).

ERα is a nuclear transcription factor that can regulate many human physiological processes (57). It has been reported that ERα can induce osteoblasts proliferation following estradiol stimulation (58). Based on this, we speculate that MGF can promote osteoblasts proliferation *via* ERα. All residues of ERα that interacted with MGF were also included by the catechin (an ERα modulator)—ERα interactions (59). The result suggested that Glu353, Leu346, Ala350, and Leu525 residues were critical for ERα-binding peptides, while Leu387, Met388, and Phe404 residues could contribute to peptides’ osteoblasts proliferation stimulation effect.

## Conclusion

This study identified three di/tripeptides with potential osteoblasts proliferation stimulation abilities from yak bone collagen, namely, MGF, CF, and CF, by *in silico* screening. Among them, MGF showed significant (*P* < 0.05) MEC3T3-E1 cells’ proliferation-promoting activities (the relative proliferation rate was 116.32%) after the treatment for 72 h at 0.3 mg/ml, which was consistent with the results of *in silico* screening. The proliferation-promoting effect of MGF may attribute to its particular binding sites with EPCR (Leu82), CBR2 (Ser90, Phe91, His95, Pro184, Ile186, Tyr190, and Leu191), and ERα (Leu387, Met388, and Phe404). The result suggested that MGF could be used as a lead compound for anti-osteoporosis drugs. However, *in vivo* assays are needed to validate the *in silico* prediction results. Other potential bioactivities of MGF are also needed for further exploration.

## Data Availability Statement

The datasets presented in this study can be found in online repositories. The names of the repository/repositories and accession number(s) can be found below: https://www.ncbi.nlm.nih.gov/, ELR46121; https://www.ncbi.nlm.nih.gov/, ELR60286.

## Author Contributions

YC: conceptualization, methodology, software, and writing. YG: conceptualization, methodology, review, and project administration. YL: methodology. CZ: funding acquisition, project administration, supervision, and review. FH and LC: conceptualization and suggestion. All authors contributed to the article and approved the submitted version.

## Conflict of Interest

The authors declare that the research was conducted in the absence of any commercial or financial relationships that could be construed as a potential conflict of interest.

## Publisher’s Note

All claims expressed in this article are solely those of the authors and do not necessarily represent those of their affiliated organizations, or those of the publisher, the editors and the reviewers. Any product that may be evaluated in this article, or claim that may be made by its manufacturer, is not guaranteed or endorsed by the publisher.

## Footnotes

1
https://www.ncbi.nlm.nih.gov/

2
http://web.expasy.org/peptide_cutter/

3
http://distilldeep.ucd.ie/PeptideRanker/

4
http://www1.rcsb.org/

5
https://www.ncbi.nlm.nih.gov/

6
https://web.expasy.org/protscale
