# Supplementary material for: Identification of Di/Tripeptide(s) With Osteoblasts Proliferation Stimulation Abilities of Yak Bone Collagen by in silico Screening and Molecular Docking
Source: Front Nutr. 2022 May 30;9:874259. doi: 10.3389/fnut.2022.874259 (PMC9197386; doi:10.3389/fnut.2022.874259)
Supplement: Supplementary file 1 [file Data_Sheet_1.PDF]

Supplementary Material

The sequence of α_1 chain of yak collagen-I

MFSFVDLRLLLLLAATALLTHGQEEGQEEGQEEDIPPVTCVQNGLRYHDRDVWKPVPCQI
CVCDNGNVLCDDVICDELKDCPNAKVPTDECCPVCPEGQESPTDQETTGVEGPKGDTGP
RGPRGPAGPPGRDGIPGQPGLPGPPGPPGPPGPPGLGGNFAPQLSYGYDEKSTGISVPGP
MGPSGPRGLPGPPGAPGPQGFQGPPGEPGEPGASGPMGPRGPPGPPGKNGDDGEAGKP
GRPGERGPPGPQGARGLPGTAGLPGMKGHRGFSGLDGAKGDAGPAGPKGEPGSPGEN
GAPGQMGPRGLPGERGRPGAPGPAGARGNDGATGAAGPPGPTGPAGPPGFPGAVGAK
GEGGPQGPRGSEGPQGVRGEPGPPGPAGAAGPAGNPGADGQPGAKGANGAPGIAGAP
GFPGARGPSGPQGPSGPPGPKGNSGEPGAPGSKGDTGAKGEPGPTGIQGPPGPAGEEGK
RGARGEPGPAGLPGPPGERGGPGSRGFPGADGVAGPKGPAGERGAPGPAGPKGSPGEA
GRPGEAGLPGAKGLTGSPGSPGPDGKTGPPGPAGQDGRPGPPGPPGARGQAGVMGFPG
PKGAAGEPGKAGERGVPGPPGAVGPAGKDGEAGAQGPPGPAGPAGERGEQGPAGSPGF
QGLPGPAGPPGEAGKPGEQGVPGDLGAPGPSGARGERGFPGERGVQGPPGPAGPRGAN
GAPGNDGAKGDAGAPGAPGSQGAPGLQGMPGERGAAGLPGPKGDRGDAGPKGADG
APGKDGVRGLTGPIGPPGPAGAPGDKGEAGPSGPAGPTGARGAPGDRGEPGPPGPAGFA
GPPGADGQPGAKGEPGDAGAKGDAGPPGPAGPAGPPGPIGNVGAPGPKGARGSAGPP
GATGFPGAAGRVGPPGPSGNAGPPGPPGPAGKEGSKGPRGETGPAGRPGEVGPPGPPGP
AGEKGAPGADGPAGAPGTPGPQGIAGQRGVVGLPGQRGERGFPGLPGPSGEPGKQGPS
GASGERGPPGPMGPPGLAGPPGESGREGAPGAEGSPGRDGSPGAKGDRGETGPAGPPG
APGAPGAPGPVGPAGKSGDRGETGPAGPAGPIGPVGARGPAGPQGPRGDKGETGEQGD
RGIKGHRGFSGLQGPPGPPGSPGEQGPSGASGPAGPRGPPGSAGSPGKDGLNGLPGPIGP
PGPRGRTGDAGPAGPPGPPGPPGPPGPPSGGYDLSFLPQPPQEKAHDGGRYYRADDANV
VRDRDLEVDTTLKSLSQQIENIRSPEGSRKNPARTCRDLKMCHSGEYWIDPNQGCNLDAI
KVFCNMETGETCVYPTQPSVAQKNWYISKNPKEKRHVWYGESMTGGFQFEYGGQGSDP
ADVAIQLTFLRLMSTEASQNITYHCKNSVAYMDQQTGNLKKALLLQGSNEIEIRAEGNSRF
TYSVTYDGCTSHTGAWGKTVIEYKTTKTSRLPIIDVAPLDVGAPDQEFGFDVGPACFL

The sequence of α_2 chain of yak collagen-I

MLSFVDTRTLLLLAVTSCLATCQCKCLQLVSGSLGKSGDRGPRGERGPPGPPGRDGDDGI
PGPPGPPGPPGPPGLGGNFAAQFDAKGGGPGPMGLMGPRGPPGASGAPGPQGFQGPPG
EPGEPGQTGPAGARGPPGPPGKAGEDGHPGKPGRPGERGVVGPQGARGFPGTPGLPGF
KGIRGHNGLDGLKGQPGAPGVKGEPGAPGENGTPGQTGARGLPGERGRVGAPGPAGA
RGSDGSVGPVGPAGPIGSAGPPGFPGAPGPKGELGPVGNPGPAGPAGPRGEVGLPGLSG
PVGPPGNPGANGLPGAKGAAGLPGVAGAPGLPGPRGIPGPVGASGATGARGLVGEPGP
AGSKGESGNKGEPGAVGQPGPPGPSGEEGKRGSTGEIGPAGPPGPPGLRGNPGSRGLPG
ADGRAGVMGPAGSRGATGPAGVRGPNGDSGRPGEPGLMGPRGFPGSPGNIGPAGKEGP
VGLPGIDGRPGPIGPAGARGEPGNIGFPGPKGPSGDPGKAGEKGHAGLAGARGAPGPD
GNNGAQGPPGLQGVQGGKGEQGPAGPPGFQGLPGPAGTAGEAGKPGERGIPGEFGLPG
PAGARGERGPPGESGAAGPTGPIGSRGPSGPPGPDGNKGEPGVVGAPGTAGPSGPSGLP
GERGAAGIPGGKGEKGETGLRGDIGSPGRDGARGAPGAIGAPGPAGANGDRGEAGPA
GPAGPAGPRGSPGERGEVGPAGPNGFAGPAGAAGQPGAKGERGTKGPKGENGPVGPT
GPVGAAGPSGPNGPPGPAGSRGDGGPPGATGFPGAAGRTGPPGPSGISGPPGPPGPAGK
EGLRGPRGDQGPVGRSGETGASGPPGFVGEKGPSGEPGTAGPPGTPGPQGLLGAPGFL
GLPGSRGERGLPGVAGSVGEPGPLGIAGPPGARGPPGNVGNPGVNGAPGEAGRDGNPG
NDGPPGRDGQPGHKGERGYPGNAGPVGAAGAPGPQGPVGPVGKHGNRGEPGPAGAV
GPAGAVGPRGPSGPQGIRGDKGEPGDKGPRGLPGLKGHNGLQGLPGLAGHHGDQGAP
GAVGPAGPRGPAGPSGPAGKDGRIGQPGAVGPAGIRGSQGSQGPAGPPGPPGPPGPPGPS
GGGYEFGFDGDFYRADQPRSPTSLRPKDYEVDATLKSLNNQIETLLTPEGSRKNPARTCRD
LRLSHPEWSSGYYWIDPNQGCTMDAIKVYCDFSTGETCIRAQPEDIPVKNWYRNSKAKK
HVWVGETINGGTQFEYNVEGVTTKEMATQLAFMRLLANHASQNITYHCKNSIAYMDEET
GNLKKAVILQGSNDVELVAEGNSRFTYTVLVDGCSKKTNEWQKTIIEYKTNKPSRLPILDI
APLDIGGADQEIRLNIGPVCFK

Supplementary Figure 1. The sequence of yak collagen-I. (Type I collagen contains two α_1 chain and one α_2 chain. The sequence of α_1 chain was shown in the left column, and α_2 chain in the right column. Glycine was marked in red to indicate the (Glycine-X-Y) repeats. Residues in grey background were telopeptides)

Supplementary Table 1. The identified di/tri-peptides of yak bone collagen-I and their bioactivity

Number	Amino acid sequence	Length	Source	Mass	Bioactivity
1	CF	2	α1, α2	268.331	0.99641
2	CV	2	α1	220.287	0.327224
3	DA	2	α1, α2	204.183	0.131047
4	DE	2	α1	262.219	0.036851
5	DI	2	α1, α2	246.263	0.140525
6	DL	2	α1	246.263	0.325534
7	DT	2	α1, α2	234.209	0.051559
8	DV	2	α1	232.236	0.046166
9	GA	2	α1, α2	146.146	0.522353
10	GE	2	α1, α2	204.183	0.110185
11	GF	2	α1, α2	222.244	0.994712
12	GI	2	α1, α2	188.227	0.521628
13	GL	2	α1, α2	188.227	0.808777
14	GV	2	α1, α2	174.2	0.182015
15	GY	2	α1	238.243	0.741592
16	KA	2	α1	217.268	0.09845
17	KT	2	α1, α2	247.294	0.036799
18	KV	2	α1, α2	245.322	0.035865
19	MF	2	α1	296.384	0.996643
20	NI	2	α1, α2	245.278	0.140706
21	NV	2	α1, α2	231.252	0.0417
22	PI	2	α1, α2	228.291	0.546777
23	PL	2	α1, α2	228.291	0.811148
24	PT	2	α1	216.237	0.249461
25	QF	2	α1, α2	293.323	0.946135
26	QL	2	α1, α2	259.305	0.2924
27	RA	2	α1, α2	245.282	0.354353
28	RL	2	α1, α2	287.362	0.626352
29	RT	2	α1, α2	275.308	0.143727

Continued Supplementary Table 1. The identified di/tri-peptides of yak bone collagen-I and their bioactivity

30	RY	2	α1	337.379	0.543741
31	SF	2	α1, α2	252.27	0.948796
32	SV	2	α1	204.226	0.052322
33	SY	2	α1	268.269	0.262363
34	CDE	3	α1	365.358	0.167379
35	CPE	3	α1	347.386	0.449781
36	DDA	3	α1	319.271	0.127455
37	DGA	3	α1	261.235	0.319406
38	DGV	3	α1	289.288	0.161525
39	DQE	3	α1, α2	390.35	0.048562
40	GDA	3	α1	261.235	0.303413
41	GGF	3	α1	279.296	0.987345
42	GKE	3	α1, α2	332.357	0.07522
43	GKT	3	α1	304.346	0.13072
44	GNL	3	α1, α2	302.33	0.549969
45	GNV	3	α1	288.304	0.141818
46	GPA	3	α1, α2	243.263	0.725277
47	GPI	3	α1, α2	285.343	0.721084
48	GPT	3	α1, α2	273.289	0.520685
49	GPV	3	α1, α2	271.316	0.471265
50	GQE	3	α1	332.313	0.099305
51	GRV	3	α1	330.387	0.327301
52	KGA	3	α1, α2	274.32	0.211735
53	KGE	3	α1, α2	332.357	0.072524
54	KGL	3	α1	316.401	0.389123
55	KKA	3	α1, α2	345.442	0.071682
56	KPV	3	α1	342.439	0.138491
57	KSL	3	α1, α2	346.427	0.146269
58	KST	3	α1	334.373	0.047944
59	MGF	3	α1	353.436	0.990723
60	MST	3	α1	337.391	0.233717

Continued Supplementary Table 1. The identified di/tri-peptides of yak bone collagen-I and their bioactivity

61	NGA	3	α1, α2	260.25	0.274534
62	NGL	3	α1, α2	302.33	0.507526
63	PGA	3	α1, α2	243.263	0.674335
64	PGE	3	α1, α2	301.299	0.321055
65	PGF	3	α1, α2	319.36	0.987422
66	PGI	3	α1, α2	285.343	0.668218
67	PGL	3	α1, α2	285.343	0.855192
68	PGT	3	α1, α2	273.289	0.517522
69	PPV	3	α1	311.381	0.522436
70	PQL	3	α1	356.422	0.527882
71	QGL	3	α1, α2	316.357	0.533495
72	QGV	3	α1, α2	302.33	0.160355
73	RGA	3	α1, α2	302.333	0.433961
74	RGE	3	α1, α2	360.37	0.160331
75	RGF	3	α1, α2	378.431	0.969527
76	RGL	3	α1, α2	344.414	0.678651
77	RGV	3	α1, α2	330.387	0.231451
78	SGE	3	α1	291.261	0.12169
79	SGL	3	α1	275.305	0.557028
80	SHT	3	α1	343.34	0.107051
81	SMT	3	α1	337.391	0.352408
82	SPT	3	α1	303.315	0.269398
83	SRL	3	α1	374.44	0.49389
84	CDF	3	α2	383.419	0.95755
85	CI	2	α2	234.313	0.660168
86	DGL	3	α2	303.315	0.525785
87	GGA	3	α2	203.198	0.636532
88	GRT	3	α2	332.36	0.382153
89	GSA	3	α2	233.224	0.272249
90	GSV	3	α2	261.278	0.138346

Continued Supplementary Table 1. The identified di/tri-peptides of yak bone collagen-I and their bioactivity

91	GT	2	α2	176.172	0.22616
92	KE	2	α2	275.305	0.025704
93	KGI	3	α2	316.401	0.225064
94	KNW	3	α2	446.506	0.659952
95	MA	2	α2	220.287	0.693293
96	MDA	3	α2	335.375	0.395826
97	MDE	3	α2	393.412	0.158947
98	ML	2	α2	262.367	0.894564
99	MRL	3	α2	418.555	0.818877
100	NE	2	α2	261.235	0.0345
101	NGT	3	α2	290.276	0.168755
102	NHA	3	α2	340.339	0.137741
103	PE	2	α2	244.247	0.145789
104	PGV	3	α2	271.316	0.427664
105	PV	2	α2	214.265	0.20792
106	QKT	3	α2	375.425	0.051506
107	QPE	3	α2	372.378	0.14439
108	RGI	3	α2	344.414	0.467663
109	RGT	3	α2	332.36	0.295709
110	RGY	3	α2	394.431	0.610633
111	SCL	3	α2	321.392	0.710595
112	SGA	3	α2	233.224	0.327516
113	SL	2	α2	218.253	0.330018
114	ST	2	α2	206.199	0.060847

Supplementary Table 2. The ADMET properties of di/-tripeptides of yak bone collagen-I and their molecular docking results (bioactivity value >0.5)

Amino acid sequence	Length	Aqueous solubility[a]	Cytochrome P4502D6 inhibition	Hepatotoxicity	HIA[b]	-CE (Kcal/mol)			
						EPCR	CBR2	ERα	Total
MF	2	4	FALSE	FALSE	0	24.2213	44.0525	44.9097	113.1835
CF	2	4	FALSE	FALSE	0	23.3183	42.6237	49.4294	115.3714
GF	2	5	FALSE	FALSE	0	24.8121	36.3484	43.4265	104.587
MGF	3	4	FALSE	FALSE	1	41.7988	52.0797	57.0684	150.9469
PGF	3	5	FALSE	FALSE	1	23.908	31.5368	42.1162	97.561
GGF	3	5	FALSE	FALSE	2	—	—	—	—
RGF	3	4	FALSE	FALSE	3	—	—	—	—
CDF	3	4	FALSE	FALSE	3	—	—	—	—
SF	2	5	FALSE	FALSE	1	24.164	43.1441	41.4631	108.7712
QF	2	5	FALSE	FALSE	2	—	—	—	—
ML	2	5	FALSE	FALSE	0	20.3414	44.5671	42.2125	107.121
PGL	3	5	FALSE	TRUE	1	—	—	—	—
MRL	3	3	FALSE	FALSE	3	—	—	—	—
PL	2	5	FALSE	TRUE	0	—	—	—	—
GL	2	5	FALSE	TRUE	1	—	—	—	—
GY	2	5	FALSE	FALSE	1	28.5544	37.4608	47.6404	113.6556
GPA	3	5	FALSE	TRUE	3	—	—	—	—
GPI	3	5	FALSE	TRUE	1	—	—	—	—
SCL	3	5	FALSE	TRUE	3	—	—	—	—
MA	2	5	FALSE	FALSE	1	21.9221	36.4377	40.3591	98.7189
PGA	3	5	FALSE	TRUE	3	—	—	—	—
PGI	3	5	FALSE	TRUE	1	—	—	—	—
CI	2	5	FALSE	TRUE	0	—	—	—	—
KNW	3	5	FALSE	TRUE	3	—	—	—	—
GGA	3	5	FALSE	TRUE	3	—	—	—	—
RL	2	4	FALSE	FALSE	3	—	—	—	—

Continued Supplementary Table 2. The ADMET properties of di/-tripeptides of yak bone collagen-I and their molecular docking results (bioactivity value >0.5)

Amino acid sequence	Length	Aqueous solubility[a]	Cytochrome P4502D6 inhibition	Hepatotoxicity	HIA[b]	-CE (Kcal/mol)			
						EPCR	CBR2	ERα	Total
RGY	3	3	FALSE	FALSE	3	—	—	—	—
SGL	3	5	FALSE	TRUE	3	—	—	—	—
GNL	3	5	FALSE	TRUE	3	—	—	—	—
PI	2	5	FALSE	TRUE	0	—	—	—	—
RY	2	4	FALSE	FALSE	3	—	—	—	—
QGL	3	5	FALSE	TRUE	3	—	—	—	—
PQL	3	5	FALSE	TRUE	2	—	—	—	—
DGL	3	5	FALSE	TRUE	3	—	—	—	—
PPV	3	4	FALSE	TRUE	0	—	—	—	—
GA	2	5	FALSE	TRUE	3	—	—	—	—
GI	2	5	FALSE	TRUE	1	—	—	—	—
GPT	3	5	FALSE	TRUE	3	—	—	—	—
PGT	3	5	FALSE	TRUE	3	—	—	—	—
NGL	3	5	FALSE	TRUE	3	—	—	—	—
RGL	3	4	FALSE	FALSE	3	—	—	—	—

[a]For solubility, “0” indicates extremely low, “1” indicates very low, “2” indicates low, “3” indicates good, “4” indicates optimal, and “5” indicates very soluble. [b]For HIA, “0” indicates good, “1” indicates moderate, “2” indicates poor, and “3” indicates very poor.
